# Development and Validation of a Commercial TaqMan-Based RT-qPCR Kit for Rotavirus and Norovirus Detection in the Brazilian Acute Diarrhea Surveillance Network

**DOI:** 10.3390/v17121559

**Published:** 2025-11-28

**Authors:** Geison Cambri, Thiago Jacomasso, Fernanda Marcicano Burlandy, Fábio Correia Malta, Alexandre Madi Fialho, Audrey Cilli, Simone Guadagnucci, Dielle Monteiro Teixeira, Patrícia Santos Lobo, Hugo Reis Resque, Lucia Helena Berto, Alessandro Afornali, Fabricio Klerynton Marchini, Irina Nastassja Riediger, Luana Silva Soares, Rita de Cássia Compagnoli Carmona, Tulio Machado Fumian

**Affiliations:** 1 Molecular Biology Institute of Paraná (IBMP), Curitiba 81350-010, PR, Brazil; geison.cambri@ibmp.org.br (G.C.); alessandro.afornali@ibmp.org.br (A.A.); fabricio.marchini@ibmp.org.br (F.K.M.); 2 Laboratory of Comparative and Environmental Virology (LVCA), Oswaldo Cruz Institute, Oswaldo Cruz Foundation (Fiocruz), Av. Brasil 4365, Rio de Janeiro 21040-360, RJ, Brazil; fburlandy@ioc.fiocruz.br (F.M.B.); fabio.malta@ioc.fiocruz.br (F.C.M.); amfialho@ioc.fiocruz.br (A.M.F.); 3 Enteric Diseases Laboratory (LDE), Virology Center (CV), Adolfo Lutz Institute (IAL), Av. Dr Arnaldo, 355, São Paulo 01246-900, SP, Brazil; audrey.chirelli@ial.sp.gov.br (A.C.); sgmorillo@gmail.com (S.G.); 4 Laboratory of Gastroenteric Viruses (LVG), Virology Section, Evandro Chagas Institute, Secretariat of Health Surveillance and Environment, Brazilian Ministry of Health, Ananindeua 67030-000, PA, Brazil; dielleteixeira@iec.gov.br (D.M.T.); patricialobo@iec.gov.br (P.S.L.); hugoresque@iec.gov.br (H.R.R.); 5 General Coordination of Public Health Laboratories (CGLab), Ministry of Health, Federal District, Brasília 70058-900, DF, Brazil; lucia.berto@saude.gov.br; 6 Laboratory for Applied Science and Technology in Health, Carlos Chagas Institute, Oswaldo Cruz Foundation (Fiocruz-PR), Curitiba 81350-010, PR, Brazil

**Keywords:** Acute gastroenteritis, rotavirus, norovirus, diagnostic, quantitative PCR

## Abstract

Acute gastroenteritis (AGE) is a major cause of illness and death in children under five, especially in low- and middle-income countries, and rotavirus A (RVA) and norovirus are the leading viral agents. The present study aimed to describe the development of a commercial multiplex TaqMan-based RT-qPCR assay to detect those viruses to enhance surveillance and public health responses in Brazil. The assay validation involved optimizing primers and probes for multiplex RT-qPCR, assessing analytical sensitivity, and confirming specificity. A multicenter pilot study across Brazil’s AGE surveillance network assessed the assay’s performance. The IBMP NAT assay demonstrated high specificity and sensitivity for detecting RVA and norovirus GI and GII. No cross-reactivity was observed. LoD95 values were low: 18.6 (GI), 71.2 (GII), and 12.3 (RVA) copies/reaction. In 379 clinical samples, diagnostic sensitivity and specificity exceeded 96% for all targets. The assay showed strong reproducibility across operators and instruments. Stability tests confirmed consistent performance under freeze–thaw, transport, and storage conditions. Compared to in-house RT-qPCR, the IBMP NAT test yielded lower Ct values, indicating improved detection of low viral loads. The IBMP NAT Kit significantly advances molecular diagnostics, enabling rapid, sensitive, and reliable detection of RVA and norovirus in fecal specimens. It strengthens public health surveillance and supports timely responses to AGE outbreaks, helping reduce disease burden in vulnerable populations.

## 1. Introduction

Acute gastroenteritis (AGE) is one of the leading causes of morbidity and mortality in children under the age of five, especially in low- and middle-income countries (LMICs), where access to healthcare, hygiene, and education is often limited [1,2]. Studies conducted in LMICs that screened for multiple enteropathogens have identified rotavirus and norovirus as the predominant enteric pathogens responsible for pediatric AGE [3,4,5,6].

*Rotavirus alphagastroenteritidis* (formerly species A rotavirus; RVA) is the leading cause of AGE in children under 5 years, posing a significant global health threat especially in LMICs [3]. In Brazil, since 2013, more than 5000 severe RVA infection cases have been officially reported to the national RVA surveillance program of the Brazilian Ministry of Health [7]. The outer capsid proteins, VP7 (a capsid glycoprotein) and VP4 (a spike protein), independently elicit neutralizing antibodies and form the basis of the binary classification system for G and P types, respectively [8]. Currently, 42 G and 58 P genotypes are recognized as capable of infecting both humans and animals (https://rega.kuleuven.be/cev/viralmetagenomics/virus-classification/rcwg, accessed on 18 January 2025); however, some genotypes, such as G1P[8], G2P[4], G3P[8], G4P[8], G9P[8], and G12P[8] are globally significant in clinical settings [9].

Noroviruses belong to the genus *Norovirus* within the *Caliciviridae* family. Noroviruses are classified into ten distinct genogroups (GI-GX) and divided into 48 genotypes, however only GI, GII, GIV, GVIII and GIX are known to infect humans [10,11]. Among these, GI and GII noroviruses are responsible for most human infections [12]. Since the introduction of the RVA vaccine in several countries, norovirus has emerged as the predominant cause of AGE, particularly in countries where the RVA vaccine has been publicly available. In those countries, norovirus-related illnesses now account for a substantial proportion of medical visits due to AGE in pediatric populations [13,14,15]. Currently, noroviruses are recognized as the leading cause of AGE across all age groups worldwide, responsible for approximately 20% of AGE episodes and over 200,000 deaths annually in low- and middle-income countries [11,16,17].

Early diagnosis of viral AGE cases plays a pivotal role, especially in preventing the unnecessary and inappropriate use of antimicrobials, as well as to evaluate the ongoing prevention strategies, such as RVA vaccination. Additionally, comprehensive screening and increasing the capacity for RVA and norovirus testing are crucial. A wide range of diagnostic methods have been employed for detecting rotavirus infection in stool samples, including commercial enzyme-linked immunosorbent assay (ELISA) and immunochromatographic tests, particularly in clinical settings. In research laboratories involved in vaccine development and epidemiological studies, molecular assays, both conventional and real-time RT-PCR, are frequently used to complement antigen detection methods. For norovirus, due to the antigenic variability, which poses challenges for high-sensitivity antigen detection, RT-qPCR remains the gold standard for detecting and quantifying the viral RNA in stool samples. In Brazil, laboratory surveillance of AGE cases is initially conducted at Central Public Health Laboratories (LACENs) in each state where RVA infection is detected using ELISA. Reference laboratories perform further diagnostic confirmation using molecular approaches to determine circulating genotypes.

Although several multiplex RT-qPCR assays for RVA and norovirus have been described previously, the IBMP-NAT (Instituto de Biologia Molecular do Paraná—Nucleic Acid Test) Rotavirus and Norovirus Kit represent the first fully validated, ready-to-use registered commercial assay specifically standardized for the Brazilian public health surveillance network. Our assay integrates (i) simultaneous detection of RVA and norovirus GI and GII genogroups with an internal endogenous control, (ii) analytical performance aligned with recommended diagnostic standards, and (iii) validation across three national Rotavirus Reference Laboratories using routine clinical stool samples. This combination ensures high diagnostic sensitivity, reproducibility, and logistical feasibility for large-scale implementation within the Central Public Health Laboratories (LACEN) system.

## 2. Materials and Methods

### 2.1. Ethical Clearance

This study received approval from the Ethics Committees of the Oswaldo Cruz Foundation (FIOCRUZ; in CAAE 76063123.5.0000.5248), the Adolfo Lutz Institute (IAL; in CAAE 91636825.1.0000.0059), and the Evandro Chagas Institute (IEC; in CAAE 92340825.7.0000.0019). The surveillance was conducted through a hierarchical network, in which samples were obtained based on medical requests from hospitals and health centers monitored by the Brazilian Unified Health System (SUS). This study was conducted within the scope of the RRRL/MoH as part of a federal public health policy for viral AGE surveillance in Brazil. Patient-informed consent was waived by the Ethical Committee, and patient data was maintained anonymously and securely.

### 2.2. Stool Samples

This study included 902 stool samples (379 and 523 samples for the analytical performance and inter-laboratory evaluations, respectively) from inpatients and outpatients (children and adults) with AGE symptoms who were treated in sentinel units or any healthcare facility unit, as outlined in Consolidation Ordinances No. 4 and No. 5, 28 September 2017, of Brazilian Ministry of Health. For instance, AGE was defined as ≥three liquid or semi liquid evacuations within a 24 h period. Stool samples were systematically sent along with clinical-epidemiological records to one of the Rotavirus Reference Laboratory that are part of the AGE surveillance network in Brazil. The surveillance network consists of one Rotavirus National Reference Center (Evandro Chagas Institute), two Rotavirus Regional Reference Laboratories (Adolf Lutz Institute and Oswaldo Cruz Institute) and states central laboratories, all overseen by the General Coordination of Public Health Laboratories of the Brazilian Ministry of Health (CGLab/MoH). In addition to RVA surveillance, the reference laboratories also conduct norovirus diagnostic testing for all received samples.

### 2.3. Viral RNA Extraction

Viral nucleic acids were extracted from 140 µL of clarified stool suspension (10% *w*/*v* in Tris-calcium buffer, pH 7.2) using the Extracta 32 automated system and the Extracta Fast DNA and RNA Viral extraction kit (Loccus, São Paulo, Brazil). The procedure, based on magnetic bead technology, was performed according to the manufacturer’s instructions. Briefly, 140 µL of clarified stool suspension, supplemented with 5 µL of proteinase K (20 mg/mL), was transferred to the extraction plate containing lysis buffer and magnetic silica particles for nucleic acid binding. The system then performed automated lyses, capture and washing steps to remove potential inhibitors, followed by elution of purified RNA in 60 µL of RNase-free elution buffer. Extracted RNA was stored at −80 °C until further analysis.

### 2.4. Development and Design of the Assay

Primers and probes used for detecting RVA and norovirus GI/GII were based on those previously described by Zeng et al. [18], Hill et al. [19] and Kageyama et al. [20], respectively. To combine these primers and probe sets and a set targeting a human gene as internal control into one multiplex reaction, concentrations for each individual set were determined using IBMP’s proprietary RT/Taq formulation and a synthetic DNA control molecule, designed to contain the three viral targets (RVA NSP3 region, Norovirus GI and GII ORF1-2 junction region). Optimal primer concentrations were evaluated using EvaGreen intercalating dye, which allowed for the inspection of secondary amplification products that could hinder reaction performance. The selected concentration for each primer pair was the one that offered the best compromise between reaction kinetics (judged by the shape of the amplification curves) and dimer/hairpin formation (based on the melting profiles of PCR products in templated and non-templated reactions). Probe concentrations were selected based on amplification kinetics in a temperature gradient. Primers and probes sequences are not fully disclosed due to commercial confidentiality.

Primers and probes in their optimized concentrations were combined in a single reaction mix. Probes were labeled with dye and quencher pairs that are compatible with most qPCR instruments. The multiplexed reactions were then validated as described below.

### 2.5. Multiplex TaqMan-Based RT-qPCR for RVA and Norovirus Detection

RT-qPCR reactions were performed using 5 µL of the extracted RNA in a final volume of 20 µL, containing 15 µL of a PCR mixture (IBMP MixFit I, IBMP, Curitiba, PR, Brazil), which includes 5 µL of IBMP mastermix 4X, 12 ng of Taq DNA polymerase (IBMP), 75 ng of reverse transcriptase enzyme (IBMP) and primers and probes (IBMP; IDT, Coralville, IA, USA; and Thermo Fisher Scientific, Waltham, MA, USA). Reactions were conducted in the ABI 7500 Real-Time PCR System (Applied Biosystems, Foster City, CA, USA), under the following thermal cycling conditions: 15 min at 50 °C, 10 min at 95 °C, 40 cycles of 15 s at 95 °C, and 1 min at 57 °C. The RT-qPCR analysis parameters were configured in the ABI 7500 software with a baseline range of 3–15 and threshold values set to 5000 for norovirus GI, 10,000 for norovirus GII, 30,000 for RVA, and 5000 for the internal control. The results were considered positive for stool clarified samples that exhibited a characteristic sigmoid curve, and when the Ct values of both the endogenous control and rotavirus or norovirus genes were ≤40. Samples were considered negative when no amplification was detected for norovirus and rotavirus, and the internal control showed amplification with a Ct value ≤ 35. All runs included both positive and negative controls using synthetic molecules. The positive controls exhibited amplification for all targets, whereas the negative controls showed amplification exclusively for the internal control.

### 2.6. Analytical Sensitivity and Specificity of Multiplex TaqMan-Based RT-qPCR Assay

The analytical sensitivity of the assay was determined using six serial five-fold dilutions of rotavirus and norovirus (GI and GII) reference samples from Vircell (catalog number: MBC026-R; Vircell, Santa Fe, Spain) and ATCC (catalog number: VR-3234SD and VR-3235SD; American Type Culture Collection, Manassas, VA, USA), respectively, with known concentrations. A minimum of 63 and maximum of 72 replicates for each dilution was tested and the limit of detection with 95% confidence (LoD95) was determined using a probit model with interpolation of detection rates and analyte concentrations in copies per reaction. The initial concentration for each virus was 40, 180 and 280 copies/reaction for norovirus GI, norovirus GII and RVA, respectively. RNA reference samples were prepared in TE buffer (pH 8.0) containing 5 µg/mL of salmon sperm DNA, 10 µg/mL of poly(A) RNA, with addition of gBlock molecule at a concentration of 10 copies/µL; gBlock was added as it contains the DNA sequence for the internal control of the multiplex reaction.

We also compared the new TaqMan-based commercial RT-qPCR assay using 379 clarified stool samples which had previously tested positive or negative. RVA- and norovirus-positive stool samples (with a wide range of Ct values) and negative samples were selected by the three RRRLs and sent to IBMP to be extracted and tested using the new multiplex protocol. Additionally, positive samples for other enteric organisms were also tested to verify the specificity.

### 2.7. Assay Repeatability, Reproducibility and Storage Stability

To assess the robustness of the TaqMan-based RT-qPCR assay, repeatability (inter-operator variability) and reproducibility (inter-thermocycler variability) were evaluated using the same dilution series from the sensitivity analysis. The dilution series consisted of six serial fivefold dilutions, with initial concentrations of 40, 180, and 280 copies/reaction for norovirus GI, norovirus GII, and rotavirus, respectively. For each concentration, a minimum of five replicates were performed per PCR run. The experiments were conducted by three independent operators using three distinct ABI 7500 Real-Time PCR Systems (Applied Biosystems, Foster City, CA, USA), with each operator performing one experiment on each thermocycler. From both assays, the mean, standard deviation (SD) and coefficient of variation (CV) were calculated independently for each target. A CV value of less than 20% for both assays was regarded as acceptable.

The stability of the new multiplex protocol was assessed through freeze–thaw cycles, transport simulation, and shelf- accelerated stability. Freeze–thaw cycles were tested by incubation of reagents at −30 to −15 °C until freezing and thawing them at room temperature. The simulation of transport was performed by incubation of reagents at −70 to −80 °C for 96 h. Finally, assessment of shelf stability was performed by an accelerated test [21,22]. Accelerated stability was calculated through a stability projection based on incubation at a stress temperature storage condition. This stability projection assumed that the product’s degradation rate decreased by a constant factor (Q10) when the storage temperature was reduced by 10 °C. The Q10 value was commonly defined in the in vitro reagent industry with acceleration factors of 2, 3, or 4, due to its activation energy under stress conditions. A Q10 value of 2, for example, provided a conservative estimate of the product’s degradation rate and was chosen in our analysis [21,22].

The formula for calculating this stability projection was defined as follows:

Q10 = X ^ΔT/10^

X = Acceleration factor.
ΔT = Temperature difference (°C) between stress conditions and ideal storage conditions (−20 °C in this case).

Thus, incubations at 4 °C for periods of 69 days were approximately equivalent to one year of incubation at −20 °C. Accordingly, 3 batches of the IBMP NAT Rotavirus and Norovirus new protocol were incubated under this condition to assess the equivalence of storage at −20 °C for 365 days.

### 2.8. Pilot Multicenter Study

A multicenter pilot study was conducted across Brazil’s national rotavirus surveillance network to evaluate the performance of the newly developed IBMP Rotavirus and Norovirus NAT test. The clinical validation involved parallel testing of 523 stool samples from patients with AGE between 2023 and 2024, using both the NAT test and established in-house RT-qPCR methods or commercial ELISA for rotavirus at three reference laboratories: IEC, National Reference Laboratory (*n* = 92); IAL, Regional Reference Laboratory (*n* = 188); and IOC, Regional Reference Laboratory (*n* = 243).

All samples were obtained through the Brazilian AGE Surveillance network from patients referred for routine diagnostic testing. The study design enabled evaluation of interlaboratory sensitivity, methodological concordance, and assay performance under standardized yet operationally diverse conditions.

### 2.9. Statistical Analysis

The Ct value for the amplification of each target gene was analyzed. The statistical significance of the differences in the different groups of data was analyzed by one-way analysis of variance (ANOVA). *p* < 0.05 was considered statistically significant. Stability comparison was performed using Repeated Measures ANOVA and *t*-tests on the Log10 (copies/reaction) values corresponding to the limit of detection for each assay, using GraphPad Prism 9.5.1 software (San Diego, CA, USA).

The Kappa coefficient was calculated to assess the level of agreement between diagnostic methods, using the reference laboratory results as the standard and including 95% confidence intervals (CI). Interpretation of Kappa values followed the criteria proposed by [23]: values between 0.81 and 1.00 indicated almost perfect agreement, 0.61–0.80 substantial, 0.41–0.60 moderate, 0.21–0.40 fair, and ≤0.20 slight agreement. Fisher’s exact test and the Chi-square test were performed.

## 3. Results

### 3.1. Multiplex Hydrolysis Probe-Based RT-qPCR Design and Analytical Sensitivity

**Figure 1:**
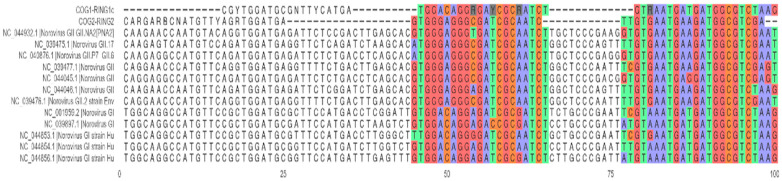
Multiple sequence alignment of COG1 and COG2 binding sites in norovirus I and II. Primer and probe sequences were concatenated to occupy a single line in the alignment. Sequences in the reverse strand (reverse primers and RING1c probe) were replaced by their reverse complement. Yellow shaded bases correspond to the align positions of primers and probes.

While adapting the reactions into a multiplex setup, we identified a cross reaction where RING1c probe (norovirus GI target) is hydrolyzed in the presence of norovirus GII RNA, resulting in an unspecific amplification signal. To investigate the causes, we evaluated the alignment of the binding regions of primers and probes to norovirus GI and GII RNAs (Figure 1).

**Figure 2:**
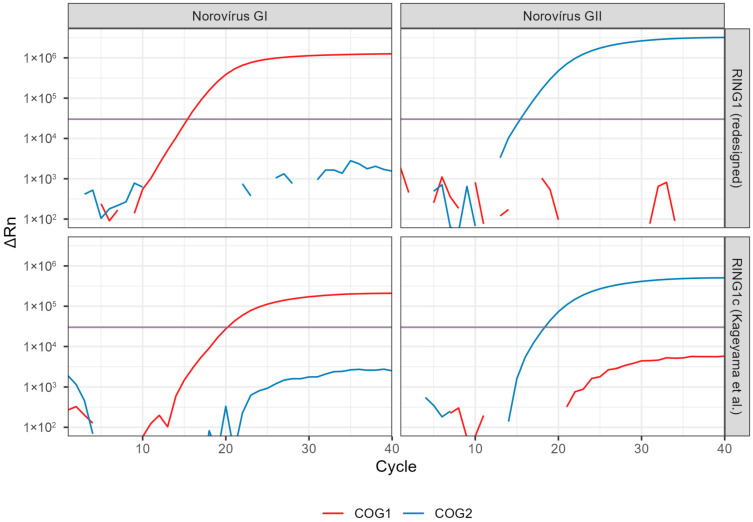
Amplification curves (ΔRn × cycle) obtained from reformulated RING1 probe versus RING1c (Kageyama et al.). Two clinical samples with high viral burden for norovirus GI (left panels) or GII (right panels) were tested with the quadruplexed reactions containing either RING1 (top panels) or RING1c (bottom panels) versions. NSP3 and for internal control are omitted for clarity. Threshold fluorescence is shown as a horizontal line. The newly designed probe displayed less unspecific signals and lower Ct values compared with the previously reported sequence.

This analysis revealed high homology between the two strains at this site. Moreover, because the probes for the norovirus GI and GII are in different strands, they are not displaced by the probe with more homology, thus allowing for both probes to be hydrolyzed (although with different efficiencies) regardless of the species in the sample. To confirm this hypothesis, we synthesized RING1, the complement to RING1c that binds to the same strand as RING2. Using both probes in the same strand improved the specificity of the signal, providing clean amplification profiles for both norovirus targets and causing no visible impact on the RVA targets (Figure 2).

After defining the optimal primer concentrations and annealing temperature (Ta), the qPCR assays were evaluated in a multiplex format, incorporating primers and a probe for the detection of a human gene as an internal control. Serial dilutions of RVA RNA (1000 to 10 copies/µL) and norovirus RNA (100 to 0.1 copies/µL) were tested, confirming specific and appropriate amplification. These results provided the basis to proceed with experiments aimed at assessing both the analytical and diagnostic sensitivity and specificity of the assay.

### 3.2. Specificity and Sensitivity

The specificity of the IBMP NAT multiplex RT-qPCR was evaluated against a panel of microorganisms, including *Clostridium difficile* (Vircell, 21MBC04001-R), *Campylobacter jejuni* (Vircell, 20MBC088001-R), *Yersinia enterocolitica* (Vircell, 20MBC027001-R), *Shigella flexneri* (Vircell, 21MBC89001-R), *Escherichia coli EAEC* (Vircell, 21MBC121001-R), *Salmonella enteritidis* (Vircell, 20MBC003001-R), *Cryptosporidium parvum* Tyzzer (ATCC, PRA-67DQ), *Entamoeba histolytica HM-1:IMSS* (ATCC, 30459D and 20459DQ), *Blastocystis hominis* strain BT1 (ATCC, 50608D), *Giardia intestinalis* WB clone C6 (ATCC, 50803D), and also clinical samples positive for sapovirus, enterovirus species A (coxsackievirus A6), enterovirus species B (echovirus 18), and adenovirus types 40 (AdVF40) and 41 (AdVF41). No cross-reactivity was detected with any of the tested organisms.

**Figure 3:**
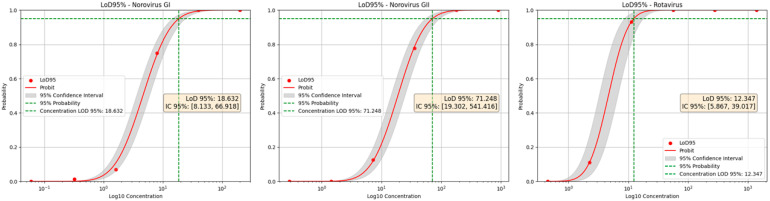
Probit analysis for 95% limit of detection of targets COG1, COG2, and NSP3. Pro-bit regression curves showing the 95% limit of detection for the targets COG1 (norovirus GI), COG2 (norovirus GII), and NSP3 (rotavirus). Red dots represent observed detection results; red lines indicate the fitted probit curves with 95% confidence intervals (shaded area). Green dashed lines mark the 95% detection probability and corresponding concentration.

Sensitivity analysis was performed based on the results of the robustness assays. To determine the Limit of Detection with 95% confidence (LoD95), six serial fivefold dilutions were prepared, starting from initial concentrations of 40, 180, and 280 copies per reaction for norovirus GI, norovirus GII, and RVA, respectively. The estimated LoD95 values were 18.6 copies/reaction for norovirus GI, 71.2 copies/reaction for norovirus GII, and 12.3 copies/reaction for RVA (Figure 3).

### 3.3. Performance Evaluation in Clinical Samples

A panel of 379 fecal suspension samples was used for the clinical performance evaluation of the IBMP NAT Rotavirus and Norovirus Kit. This panel consisted of samples positive for RVA, norovirus (GI and GII), and negative samples that tested negative for all three viruses but presented with similar clinical symptoms. The samples were provided by the three Brazilian Reference Laboratories. In total, the panel included 137 negative samples, 80 positive samples for RVA, 71 positive samples for norovirus GI, and 96 positive samples for norovirus GII. Also, five samples that were co-detected with more than one of the target viruses.

For the RVA target, the IBMP NAT Rotavirus and Norovirus Kit demonstrated a diagnostic sensitivity and specificity of 98.75% and 96.32%, respectively. The Positive Predictive Value (PPV) was 87.78%, while the Negative Predictive Value (NPV) was 99.65%. The detection of the norovirus GI demonstrated a diagnostic sensitivity and specificity of 98.59% and 98.7%, respectively. The PPV was 94.59%, and the NPV was 99.67%. For the norovirus GII target, the IBMP NAT Rotavirus and Norovirus Kit showed a diagnostic sensitivity and specificity of 97.92% and 99.29%, respectively, and the PPV was 97.92%, and the NPV was 99.29% (Table 1).

### 3.4. Robustness and Stability of the RT-qPCR Diagnostic Kit

To evaluate inter-operator reproducibility, three independent operators performed amplification assays targeting COG1, COG2, and NSP3 using serial dilutions of known input concentrations. For each target and concentration, the mean Ct value, standard deviation, and relative standard deviation (RSD%) were calculated. The results demonstrated high reproducibility among operators, with RSD values up to 5.79%, even at lower concentrations (Supplementary Table S1). These findings support the analytical consistency of the assay when performed by different users under the same conditions.

Intra-assay reproducibility of the IBMP NAT assay was evaluated using serial dilutions of known input concentrations for the targets COG1, COG2, and NSP3. Each condition was tested in replicates by three independent operators, each using three different real-time PCR thermal cyclers. For every target and concentration, mean Ct values, standard deviations, and relative standard deviations (RSD%) were calculated. For most tested concentrations, RSD values remained below 3%, confirming strong reproducibility. The highest variability was observed at the lowest input level for COG1 (1.6 copies/µL), with an RSD of 5.23%, which is expected due to increased stochastic variation at low template concentrations (Supplementary Table S2). These findings demonstrate the robustness and precision of the assay across different instruments and operators, reinforcing its suitability for routine diagnostic applications.

Stability of the IBMP NAT assay was evaluated through three experimental approaches: freeze—thaw cycling, simulated transport, and accelerated stability at 4 °C. For each condition, the 95% limit of detection (LoD95%) was determined in Log10 copies per reaction for the three assay targets (COG1, COG2, and NSP3) across three different production lots (EXT 012/24, EXT 015/24, and EXT 023/24). Statistical analyses were conducted using repeated-measures ANOVA and Student’s *t*-tests to assess intra- and inter-lot consistency and the impact of stress condition. In the freeze–thaw test, no significant differences were observed in LoD95% values between the first and fifth freeze–thaw cycles for any of the targets (COG1, COG2, NSP3), with *p*-values ranging from 0.55 to 0.96. The consistency across lots was confirmed by high inter-lot *p*-values (COG1 = 0.87; COG2 = 0.87; NSP3 = 0.42), and low t-values indicated no statistically meaningful shift (Supplementary Table S3). Similarly, in the simulated transport test, no significant changes were detected in LoD95% before and after simulated transport for any of the targets. *p*-values for COG1, COG2, and NSP3 were 0.97, 0.82, and 0.51, respectively. This indicates that the assay maintains its analytical sensitivity under physical stress conditions mimicking transportation scenarios (Supplementary Table S3).

The accelerated stability test, simulating 12 months of storage at 4 °C, also showed no statistically significant degradation in performance. For all targets, LoD95% value remained stable, with *p*-values above the significance threshold (COG1 = 0.57; COG2 = 0.25; NSP3 = 0.16). Although a slight increase in variability was observed for the NSP3 target (t = 2.10), it did not reach statistical significance (Supplementary Table S3). Overall, these results support the robustness of the IBMP NAT assay under routine handling and storage conditions. The assay exhibited consistent performance across production lots and maintained its sensitivity following environmental stress simulations.

### 3.5. Multicenter Evaluation of the IBMP NAT TaqMan-Based RT-qPCR Assay in Clinical Samples

To evaluate the performance of the IBMP NAT test TaqMan-based RT-qPCR protocol at the three reference laboratories, a total of 523 clinical samples, previously tested positive for RVA, norovirus GI or GII during routine diagnosis, were used for the analysis. Previous negative samples were also included as negative controls. The IBMP NAT test was compared to the diagnostic methodologies used at each site. Both IEC and IOC laboratories employed in-house TaqMan-based RT-qPCR assays to detect RVA and norovirus GI and GII. At IAL, RVA was detected using the Ridascreen^®^ Rotavirus ELISA (R-Biopharm, Darmstadt, Germany), while norovirus GI and GII was detected using the same in house TaqMan-based RT-qPCR assay.

At IAL, the NAT test demonstrated high concordance with ELISA for RVA detection. Among ELISA-positive samples (*n* = 115), the NAT assay detected RVA in 95.6% (*n* = 110). Of 73 ELISA-negative samples, 61 (83.5%) were also negative by NAT assay. Notably, the IBMP NAT test identified RVA in 12 ELISA-negative samples (16.4%), all of which showed high Ct values (Ct > 31), including six samples with Ct between 31 and 33, and five with Ct > 33. For norovirus GI detection, the IBMP NAT assay detected 22 of 29 positive samples (75.9%) identified by in-house RT-qPCR. Among the seven undetected samples, six had Ct values > 33. Both assays showed 100% concordance for the 159 norovirus GI-negative samples. Regarding norovirus GII, the IBMP NAT assay detected 37 of 42 positive samples (88.1%) identified by in-house RT-qPCR. The five undetected samples included three with Ct > 38. Among 146 negative samples, three were positive by IBMP NAT test (2%), though two of which had Ct values > 34.

At IEC and IOC laboratories, the performance of the IBMP NAT test was compared against in-house RT-qPCR protocols. At IEC, complete concordance (100%) was observed for RVA detection in 42 samples using both methods. The NAT assay additionally identified 10 RVA-positive samples among those previously negative by in-house RT-qPCR, all exhibiting high Ct values (>33), suggesting low viral loads. For norovirus GI, a 100% concordance was achieved between methods across all tested samples (4 positive and 88 negative). Regarding norovirus GII, the NAT assay detected 28 of 33 samples previously identified as positive by in-house RT-qPCR, corresponding to a sensitivity of 84.8%. The five undetected samples all exhibited high Ct values (>36) in the in-house assay. Both assays demonstrated 100% concordance for GII-negative samples (*n* = 59).

At IOC, 100% concordance was observed for RVA detection in 85 samples using both methods. Among 158 negative samples, the IBMP NAT test detected RVA in six samples, all with high Ct values (Ct > 32), including two samples with Ct values between 32 and 33, and four with Ct > 33. For norovirus GI, 100% concordance was achieved between methods across all tested samples (23 positive and 202 negative). Regarding norovirus GII, 100% concordance was observed in 61 samples using both methods. Among 164 negative samples, the IBMP NAT assay yielded six positive results (3.6%), with Ct values ranging from 15 to 31.

Overall, the IBMP NAT test demonstrated high sensitivity, detecting RVA in 98% (238/242) of samples previously identified as positive by in-house RT-qPCR. Additionally, the assay identified RVA in 28 samples that were negative by ELISA or RT-qPCR (10.1% of 278), though those discordant samples predominantly exhibited high Ct values (>33), suggesting lower viral loads. The overall concordance between methods for RVA detection was 90% (kappa value of 0.9 (95% CI, 0.86–0.93) (Table 2). The IBMP NAT test demonstrated 87.5% sensitivity (49/56) for norovirus GI detection, with six of seven undetected samples exhibiting high Ct values (>33). The assay showed perfect specificity (100%, 449/449) for norovirus GI-negative samples. For norovirus GII, the IBMP NAT test achieved 92.6% sensitivity (126/136), with 80% of undetected samples (8/10) showing high Ct values (Ct > 36). Among the 360 negative samples, the IBMP NAT assay yielded nine positive results (2.5%). The overall kappa values of norovirus GI and GII for the IBMP NAT test compared to the in-house RT-qPCR were 0.93 (95% CI, 0.88–0.98) and 0.92 (95% CI, 0.88–0.96), respectively (Table 2).

Concordance values between the IBMP NAT and in-house RT-qPCR assays, as well as sensitivity and sensibility values obtained in each reference laboratory for each RVA, norovirus GI and GII are presented in Table 2 and Table S4.

**Figure 4:**
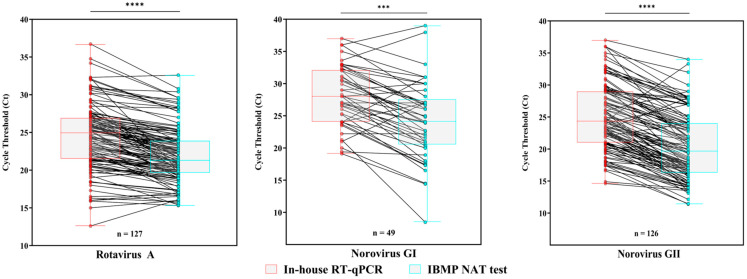
**Distribution of Cycle threshold (Ct) values obtained from in-house RT-qPCR and IBMP NAT test for rotavirus A (RVA), norovirus GI and GII positive samples tested across the three reference laboratories.** Box-and-whisker plots show the first and third quartiles (equivalent to the 5th and 95th percentiles), and the median (the horizontal line in the box). *** *p* = 0.0005; **** *p* ≤ 0.0001.

We also compared the Ct values for RVA, norovirus GI and GII obtained using both methodologies across the three reference laboratories. Overall, the IBMP NAT test detected all three viruses with lower Ct values compared to the in-house RT-qPCR. For RVA, among 126 positive samples tested, Ct values ranged from 12.6 to 36.7 and from 16.1 to 30.7 with the in-house RT-qPCR and the IBMP NAT test, respectively. The IBMP NAT test yielded significantly lower Ct values (median of 21.3) compared to the in-house RT-qPCR (median of 24.9) (*p* < 0.0001). Regarding norovirus GI, 49 samples were detected by both assays across the three reference laboratories. Ct values ranged from 19.1 to 37 with the in-house RT-qPCR and from 14.4 to 33 with the IBMP NAT test. Again, the IBMP NAT test produced significantly lower Ct values (median of 24.1) compared to the in-house assay (median of 28) (*p* = 0.0005). For norovirus GII, a similar downward trend was observed, with the IBMP NAT test yielding lower Ct values. Among the positive samples, Ct values ranged from 14.6 to 37 with the in-house RT-qPCR and from 13.5 to 34 with the IBMP NAT test. The median Ct values were 24.3 and 19.6, respectively (*p* < 0.0001) (Figure 4).

## 4. Discussion

The global implementation of RVA vaccines from 2006 onward, and their subsequent widespread use have significantly reduced RVA disease burden, as evidenced by numerous impact assessments and vaccine effectiveness studies worldwide [24,25,26]. Despite these advances, RVA remains a major cause of AGE, even with substantial declines in RVA-associated AGE cases globally [3,27,28]. For instance, a recent vaccine effectiveness study demonstrated that RVA was the leading cause of severe AGE in both vaccinated and unvaccinated children in India [29], aligning with findings from a prior ROTAVAC efficacy trial reanalysis [30]. Similarly, a Tanzanian study on ROTARIX introduction observed reduced diarrhea admissions, yet RVA persisted as the primary pathogen driving hospitalizations in children under five [25]. Noroviruses also figure as a major cause of AGE in people of all ages worldwide. In countries where the RVA vaccine has been implemented, cases of norovirus-related illnesses frequently rank as the primary reason for medical visits due to AGE in pediatric populations [13,17,26]. Together, these pathogens are responsible for millions of cases annually, imposing a significant economic and social impact on healthcare systems.

In the present study, we describe the development and evaluation of a NAT kit able to detect RVA, norovirus GI and GII, and an internal control in stool samples from AGE patients. The PCR primers were selected from a highly conserved region of the RVA non-structural protein 3 (NSP3) sequence, while for norovirus, primers and probes target a highly conserved junction region between ORFs1 and 2. Molecular diagnostics play a critical role in addressing these challenges, as conventional methods such as antigen-based assays or electron microscopy lack the sensitivity, specificity, and throughput required for reliable detection and large-scale surveillance. For instance, some studies have demonstrated that molecular methods, especially real time-based methods, are more sensitive than antigen-detection EIA assays and conventional RT-PCR [31,32]. Real-time PCR, especially in multiplex formats, provides rapid, highly sensitive, and specific results, allowing simultaneous detection of multiple targets in a single reaction. By developing a standardized kit, rather than relying on in-house protocols, issues related to inter-laboratory variability and assay validation can be minimized, thereby facilitating scalability and incorporation of molecular testing into national surveillance programs.

From a public health perspective, the availability of a national validated RT-qPCR kit contributes not only to clinical diagnosis but also to the early detection of outbreaks, and emergence epidemic virus genotypes, which is crucial for the implementation of timely control measures. Furthermore, routine surveillance supported by molecular tools enables the monitoring of viral circulation patterns, seasonality, and genotype distribution, all of which are essential to inform vaccination strategies and evaluate their long-term effectiveness. In addition, harmonized detection systems strengthen international data comparability, enhancing the capacity of global networks to respond to emerging threats. Multiple surveillance studies conducted across Brazilian regions have consistently identified RVA and norovirus as major etiological agents of AGE. For instance, in Belém, Northern Brazil, norovirus showed high prevalence (~25% positivity) among children hospitalized with AGE [33]. Similarly, a study in Rio Grande do Sul, Southern Brazil, attributed approximately 50% of AGE outbreaks to norovirus infections [34]. More recently, Sarmento et al. [35] reported a high norovirus prevalence (37.2%) among both outpatients and inpatients with AGE, during a four-year study (2019–2022) with samples from several Brazilian states. Moreover, multiple viral AGE outbreaks have been documented in Brazil, revealing diverse transmission sources. Early investigations analyzed foodborne transmission [36] and clinical cases [37], and both sample types of samples to definitively confirm outbreak sources [38]. More recent reports described outbreaks linked to commercial ice pops [39], outbreak at hospital setting with norovirus and RVA co-circulation [40], and outbreaks linked to emergent strains, including a rare GII.10[P16] recombinant [41,42]. In 2024, a major AGE outbreak involving over 217,000 cases across 180 municipalities in Goiás, Brazil, has been primarily attributed to RVA and norovirus infections, with approximately 57,000 cases reported in August alone (https://g1.globo.com/go/goias/noticia/2024/09/18/surto-de-diarreia-aguda-atinge-mais-de-180-cidades-em-goias-diz-secretaria-de-saude.ghtml, accessed on 7 October 2025). More recently, during the summer and rainy season in the Southern Hemisphere, between 29 December 2024 and 6 March 2025, a large AGE outbreak attributed to norovirus occurred in the coastal region of São Paulo State, Brazil. According to the Notifiable Diseases Information System (SINAN) of the Brazilian Ministry of Health, 54 outbreaks were reported, involving more than 76,000 cases requiring medical care, underscoring the substantial public health impact of norovirus-associated gastroenteritis in the region (https://g1.globo.com/sp/sao-paulo/noticia/2025/01/08/instituto-adolfo-lutz-confirma-presenca-de-norovirus-em-amostras-de-fezes-coletadas-na-baixada-santista-apos-casos-de-viroses.ghtml, accessed on October 7 2025).

The establishment of a real-time PCR kit targeting RVA and norovirus provides a valuable platform for both diagnostic and epidemiological applications. Its integration into disease surveillance systems, supported by the strategic use of nationally produced reagents, can significantly enhance the ability to monitor disease dynamics, improve outbreaks response, and ultimately reduce the burden associated with viral gastroenteritis, while simultaneously reinforcing the autonomy and sustainability of Brazil’s public health infrastructure.

## 5. Conclusions

The IBMP NAT Rotavirus and Norovirus Kit represents a significant advancement in molecular diagnostics, offering high sensitivity, specificity, and reproducibility for detecting RVA and norovirus G I and GII. Its low limits of detection enable accurate identification of infections even at minimal viral loads, which is crucial for early diagnosis and outbreak control. The use of these highly conserved target regions provides strong robustness and long-term sustainability for molecular surveillance of different genotypes. Additionally, its superior analytical sensitivity compared to conventional RT-qPCR methods enhances diagnostic confidence, reduces false negatives, and supports more effective clinical decision-making and public health surveillance. The kit development strengthens diagnostic capacity, enables rapid outbreak response, and will enhance nationwide surveillance of RVA and norovirus within the Brazilian Surveillance Network. In conclusion, the development of a national kit marks a strategic advance for Brazil’s health autonomy, reducing dependence on imported reagents and ensuring a sustainable, locally validated diagnostic supply. By guaranteeing continuous testing capacity even amid global disruptions, this initiative reinforces diagnostic excellence and strengthens autonomous and resilient national surveillance for RVA and norovirus.

## Figures and Tables

**Table 1:** **Performance of the molecular assay developed by IBMP for the detection of RVA, norovirus genogroup GI, and norovirus genogroup GII, compared to the reference method.** The table presents absolute concordance values (detected/not detected), total number of samples tested, sensitivity, specificity, positive predictive value (PPV), and negative predictive value (NPV), along with their respective 95% confidence intervals (95% CI).

**Rotavirus (RVA)**							
		Reference				%	95% CI
		Present	Absent	Total	Sensitivity	98.75	92.27–99.93
**IBMP**	Positive	79	11	90	Specificity	96.32	93.32–98.05
	Negative	1	288	289	PPV	87.78	78.77–93.45
	Total	80	299	379	NPV	99.65	97.78–99.98
**Norovirus GI**							
		Reference				**%**	95% CI
		Present	Absent	Total	Sensitivity	98.59	91.35–99.93
**IBMP**	Positive	70	4	74	Specificity	98.7	96.48–99.58
	Negative	1	304	305	PPV	94.59	86.02–98.25
	Total	71	308	379	NPV	99.67	97.90–99.98
**Norovirus GII**							
		Reference				%	95% CI
		Present	Absent	Total	Sensitivity	97.92	91.96–99.64
**IBMP**	Positive	94	2	96	Specificity	99.29	97.19–99.88
	Negative	2	281	283	PPV	97.92	91.96–99.64
	Total	96	283	379	NPV	99.29	97.19–99.88

**Table 2:** **Comparison of IBMP NAT test and in-house RT-qPCR results using clinical stool samples from the three Reference Laboratories**.

	General				
	Kappa 95% CI	Sensitivity (%) 95% CI	Specificity (%) 95% CI	+PV (%) ^a^ 95% CI	−PV (%) ^b^ 95% CI
Rotavirus	0.9 (0.86–0.93)	98.76 (96.42–99.74)	91.37 (87.43–94.39)	90.87 (87.17–93.59)	98.83 (96.49–99.62)
Norovirus GI	0.93 (0.88–0.98)	87.93 (76.70–95.01)	100 (99.20–100)	100 (93.02–100)	98.51 (97.05–99.25)
Norovirus GII	0.92 (0.88–0.96)	93.43 (87.90–96.95)	98.17 (96.27–99.26)	94.81 (89.76–97.45)	97.66 (95.69–98.74)

^a^ Positive predictive value; ^b^ negative predictive value.

## Data Availability

The datasets generated and analyzed during the current study are not publicly available because they contain proprietary information related to the product design and development, which is protected under industrial confidentiality agreements. Data may be available from the corresponding author from IBMP upon reasonable request and subject to approval by the company’s confidentiality policies.

## Supplementary Materials

The following supporting information can be downloaded at: https://www.mdpi.com/article/10.3390/v17121559/s1, Table S1: Repeatability assessment of the molecular assay conducted by three independent operators (Operator 1, Operator 2, and Operator 3) using serial dilutions of input concentrations for targets COG1, COG2, and NSP3. Table S2: Repeatability analysis of the qPCR assay using a 5-fold serial dilution of input concentrations (copies/µL) for the targets COG1, COG2, and NSP3. Table S3: Summary of LoD95% (Log10 copies/reaction) for COG1, COG2, and NSP3 targets across three IBMP NAT assay lots (EXT 012/24, EXT 015/24, EXT 023/24) under freeze–thaw cycling, simulated transport, and accelerated 12-month storage at 4 °C. Table S4: Comparison of IBMP NAT test and in-house RT-qPCR results using clinical stool samples discriminated by the three Reference Laboratories.

## Abbreviations

The following abbreviations are used in this manuscript:

AGE	Acute gastroenteritis
CV	coefficient of variation
dsRVA	double-stranded RNA
ELISA	enzyme-linked immunosorbent assay
IBMP NAT	Instituto de Biologia Molecular do Paraná—Nucleic Acid Test
LACEN	Central Public Health Laboratory
LMIC	low- and middle-income countries
ORF	open reading frames
Q10	constant factor
RdRp	RNA-dependent RNA polymerase
RSD%	relative standard deviations
RRRL/MoH	National Network of Reference Laboratories for Rotavirus/Ministry of Health
SD	standard deviation
SUS	Brazilian Unified Health System
