# Supplementary material for: Development and Validation of a Commercial TaqMan-Based RT-qPCR Kit for Rotavirus and Norovirus Detection in the Brazilian Acute Diarrhea Surveillance Network
Source: Viruses. 2025 Nov 28;17(12):1559. doi: 10.3390/v17121559 (PMC12737532; doi:10.3390/v17121559)
Supplement: Supplementary file 1 [file viruses-17-01559-s001.zip › viruses-3970478-supplementary.pdf]

Supplementary Table S1. **Repeatability assessment of the molecular assay conducted by three independent operators (Operator 1, Operator 2, and Operator 3) using serial dilutions of input concentrations for targets COG1, COG2, and NSP3.** For each concentration (copies/µL), the table shows the mean cycle threshold (Ct), standard deviation, and relative standard deviation (RSD%), evaluating intra-operator variability and consistency across different concentration levels.

Operator 1

Target	Input Concentration (copies/µL)	Mean Ct	Standard Deviation	RSD (%)	n (replicates)
COG1	40	28,37	0,43	1,5	21
COG1	8	30,75	0,45	1,46	24
COG1	1,6	33,58	1,8	5,35	24
COG2	180	27,29	0,4	1,46	21
COG2	36	29,67	0,47	1,58	24
COG2	7,2	32,62	1,22	3,74	24
NSP3	280	27,94	0,23	0,84	21
NSP3	56	29,83	0,19	0,65	24
NSP3	11,2	31,72	0,28	0,88	24
NSP3	2,24	35,05	1,57	4,47	24

Operator 2

Target	Input Concentration (copies/µL)	Mean Ct	Standard Deviation	RSD (%)	n (replicates)
COG1	40	28,47	0,48	1,68	21
COG1	8	31,28	1,59	5,09	24
COG1	1,6	34,63	2	5,79	24
COG2	180	27,48	0,41	1,51	21
COG2	36	30,23	1,25	4,15	24
COG2	7,2	32,5	0,63	1,93	24
NSP3	280	27,78	0,52	1,87	21
NSP3	56	29,77	0,39	1,32	24
NSP3	11,2	31,89	0,31	0,98	24
NSP3	2,24	35,36	1,29	3,64	24

Operator 3

Target	Input Concentration (copies/µL)	Mean Ct	Standard Deviation	RSD (%)	n (replicates)
COG1	40	28,52	0,5	1,76	21
COG1	8	30,82	0,52	1,69	24
COG1	1,6	34,57	1,57	4,54	24
COG2	180	27,23	0,23	0,86	21
COG2	36	29,58	0,22	0,74	24
COG2	7,2	32,53	0,48	1,48	24
NSP3	280	28,17	0,24	0,87	21
NSP3	56	30,15	0,18	0,6	24
NSP3	11,2	32,23	0,22	0,69	24
NSP3	2,24	36,35	1,73	4,77	24

Supplementary Table S2. Repeatability analysis of the qPCR assay using a 5-fold serial dilution of input concentrations (copies/µL) for the targets COG1, COG2, and NSP3. For each target and dilution point, the mean quantification cycle (Cq), standard deviation (SD), and relative standard deviation (RSD%) are presented, providing a measure of intra-assay precision across the tested dynamic range.

Target	Input Concentration (copies/µL)	Mean Ct	Standard Deviation	RSD (%)	n (replicates)
COG1	40	28,45	0,47	1,65	63
COG1	8	30,95	0,85	2,76	72
COG1	1,6	34,26	1,79	5,23	72
COG2	180	27,33	0,35	1,28	63
COG2	36	29,83	0,65	2,17	72
COG2	7,2	32,55	0,78	2,39	72
NSP3	280	27,96	0,33	1,19	63
NSP3	56	29,92	0,26	0,86	72
NSP3	11,2	31,95	0,27	0,85	72
NSP3	2,24	35,58	1,53	4,29	72

Supplementary Table S3. Summary of LoD95% (Log10 copies/reaction) for COG1, COG2, and NSP3 targets across three IBMP NAT assay lots (EXT 012/24, EXT 015/24, EXT 023/24) under freeze–thaw cycling, simulated transport, and accelerated 12-month storage at 4 °C. Statistical analysis was performed using repeated-measures ANOVA and Student's t-test to compare lot-to-lot and pre/post-condition values.

Condition	Target	EXT 012/24	EXT 015/24	EXT 023/24	ANOVA p-value	T-test p-value	T value
Freeze–Thaw (Cycle 1)	COG1	0,45	1,39	0,45	0,87	0,96	0,05
Freeze–Thaw (Cycle 5)	COG1	1	0,38	1	0,87	0,96	0,05
Simulated Transport (Validation)	COG1	0,45	1,39	0,45	0,8	0,97	0,04
Simulated Transport (Post-Transport)	COG1	0,91	0,45	1	0,8	0,97	0,04
Accelerated Stability (4 °C)	COG1	0,69	1,09	1,04	0,36	0,57	0,67
Freeze–Thaw (Cycle 1)	COG2	1,29	1,6	1,08	0,87	0,67	0,5
Freeze–Thaw (Cycle 5)	COG2	1,6	1,19	1,6	0,87	0,67	0,5
Simulated Transport (Validation)	COG2	1,29	1,6	1,08	0,74	0,82	0,25
Simulated Transport (Post-Transport)	COG2	0,99	1,08	1,65	0,74	0,82	0,25
Accelerated Stability (4 °C)	COG2	1	1,04	1,11	0,57	0,25	1,6
Freeze–Thaw (Cycle 1)	NSP3	0,6	1,27	1,09	0,42	0,56	0,69
Freeze–Thaw (Cycle 5)	NSP3	0,57	0,57	1,27	0,42	0,56	0,69
Simulated Transport (Validation)	NSP3	0,6	1,27	1,09	0,4	0,51	0,78
Simulated Transport (Post-Transport)	NSP3	0,48	0,55	1,3	0,4	0,51	0,78
Accelerated Stability (4 °C)	NSP3	0,44	0,44	0,79	0,43	0,16	2,1

Supplementary Table S4. Comparison of IBMP NAT test and in-house RT-qPCR results using clinical stool samples discriminated by the three Reference Laboratories.

	Adolfo Lutz Institute			Evandro Chagas Institute			Oswaldo Cruz Institute		
	Kappa 95% CI	Sensitivity (%) 95% CI	Specificity (%) 95% CI	Kappa 95% CI	Sensitivity (%) 95% CI	Specificity (%) 95% CI	Kappa 95% CI	Sensitivity (%) 95% CI	Specificity (%) 95% CI
**Rotavirus**	0.84<br>0.76 – 0.92	97.37<br>91.93 – 99.32	84.72<br>73.88 – 91.77	0.78<br>0.66 – 0.91	100<br>89.79 – 100	79.59<br>65.24 – 89.28	0.97<br>0.94 – 1.00	100<br>94.6 – 100	98.09<br>94.08 – 99.5
**Norovirus GI**	0.84<br>0.73 – 0.96	75.86<br>56.08 – 88.98	100<br>97.02 – 100	1<br>1.00 – 1.00	100<br>39.58 – 100	100<br>94.79 – 100	1<br>1.00 – 1.00	100<br>83.42 – 100	100<br>97.83 – 100
**Norovirus GII**	0.89<br>0.81 – 0.97	90.24<br>75.94 – 96.83	97.93<br>93.60 – 99.46	0.88<br>0.77 – 0.98	84.85<br>67.33 – 94.28	100<br>92.38 – 100	0.96<br>0.92 – 1.00	100<br>92.84 – 100	97.77<br>94 – 99.28
